# Role of MEK1 in TLR4 Mediated Signaling

**DOI:** 10.4172/2576-1471.1000135

**Published:** 2017-02-13

**Authors:** Christian Bauerfeld, Lobelia Samavati

**Affiliations:** 1 Department of Pediatrics, Division of Critical Care, Wayne State University School of Medicine and Children’s Hospital of Michigan, Detroit, USA; 2 Department of Medicine, Division of Pulmonary, Critical Care and Sleep Medicine, Wayne State University School of Medicine and Detroit Medical Center, Detroit, USA; 3 Center for Molecular Medicine and Genetics, Wayne State University School of Medicine, Detroit, USA

## Commentary

The highly evolutionary conserved mitogen activated protein kinase kinases 1 and 2 (MEK1/2), are known as upstream protein kinases that phosphorylate ERK1and ERK2. There are two distinct gene isoforms *Mek1* and *Mek2*. While MEK1 and MEK2 show high homology in their kinase domains, their N termini are significantly divergent. The differential role of *MEK1* and *MEK 2* in cellular signaling in inflammation has not been widely investigated. In this commentary, we address new insights gained in recent years and comment on emerging work using a genetic approach to address the role of MEK1 in TLR4 mediated signaling as well as possible alternative pathways that lead to ERK activation in macrophages independent of MEK1.

The mitogen activated protein kinase kinase (MAP2K) 1 and 2, also known as MEK1/2, are protein kinases that phosphorylate serine/threonine residues on extracellular signal–regulated kinases (ERK) 1 and 2, hence increasing their activity. Upstream of MEK, the RAS-RAF pathway is one of the most extensively studied signal transduction pathways and has long been implicated in the transmission of extracellular signals such as growth factors through GTPase membrane bound receptors followed by a cascade of intracellular signaling to their respective nuclear targets [1]. The physiological consequences of MEK/ERK activation depend on the extracellular stimuli, cell type and pathways involved as well as the interaction with certain scaffolding proteins and the subcellular distribution of ERK1/2 [2,3]. This can lead to a variety of outcomes including proliferation, oncogenesis, cell differentiation and cell cycle regulation. It is not surprising that with overwhelming frequency the MEK/ERK pathways are aberrantly regulated in various cancers promoting inappropriate cell proliferation, survival and metastasis [4,5].

Additionally, members of the RAS-RAF-MEK-ERK pathway can interact with kinases from other pathways, especially with the PI3K/PTEN/Akt/mTOR and Jak/STAT pathways, to modulate their activity [6]. For instance, MEK1 can regulate AKT phosphorylation through recruitment of phosphatase and tensin homolog (PTEN) [7]. Previously, we have shown that low dose rapamycin, a well-known inhibitor of the mTOR pathway, activates the MEK/ERK and AKT pathways and this was required for the induction of dual specificity phosphatase-1 (DUSP-1 or MKP-1) [8]. MKP-1 is an important phosphatase, which regulates de-phosphorylation of several MAP kinases, including p38, JNK as well as ERK. In that study, both MEK1 and MEK2 deficient macrophages failed to respond to rapamycin with MKP-1 upregulation [8]. Another member of the serine–threonine kinases that is evolutionary related to phosphatidylinositol 3-kinase related kinase (PIKKs), is ataxia-telangiectasia mutated (ATM) [9]. ATM has been implicated in activation of ERK in response to DNA damage [10]. One study suggested that ATM mutated lung carcinomas are highly susceptible to MEK1/2 inhibitor treatment. This was thought to be related to the known cross talk between the MAPK and AKT/mTOR signaling pathways, which leads to an increased dependency on MEK kinase activity for cell survival due to the inability of these cells to compensate through the pro-survival AKT/mTOR pathway [11]. These findings collectively suggest an intensive cross talk between the PI3K/AKT/mTOR and the MEK/ERK pathways.

In mammalian cells there are two distinct gene isoforms *Mek1* and *Mek2*, which are highly evolutionary conserved [12,13]. The only known downstream substrates of MEK1/2 are the ERK isoforms ERK1 and ERK2. In this regard MEK1/2 is exquisitely specific, whereas ERK can activate hundreds of substrates [14]. While MEK1 and MEK2 show high homology in their kinase domains, their N termini and their proline-rich domains are significantly divergent (only 40% identity), which provides an opportunity for the two isoform to interact and partner differently with scaffolding proteins as well as their activators and substrates [15,16]. This may provide an explanation of the distinctive role of these kinases in cellular function, in contrast to the common assumption that these two isoforms are functionally equivalent.

For instance, the deletion of the *Mek1* gene leads to embryonic lethality most likely due to aberrant angiogenesis of the placenta, whereas the interruption of the *Mek2* gene is compatible with life [17,18]. MEK1 has a regulatory role in cell migration [18,19]. Interestingly, MEK1 deficient mice exhibit a lupus-like syndrome and myeloproliferative phenotype [7]. There is evidence that MEK1 and MEK2 play a unique role in ERK activation depending on cell types and signal [7,20]. For instance, one study has shown that MEK1 deficient fibroblasts respond to EGF stimulation with a sustained ERK1/2 phosphorylation and this prolonged ERK activation was due to a lack of a negative feedback loop through MEK1/MEK2 heterodimerization [20].

There is limited data available about the role of MEK2. One study found that MEK2 is the predominant isoform in human neutrophils, and MEK2 exhibited considerably higher activity than MEK1 in response to chemotactic peptide [21].

In contrast to the well-defined role of the MEK/ERK pathway in cancer biology, the roles of MEK1 and MEK2 in response to Toll like receptor (TLR) activation is not well understood. TLRs are type I transmembrane proteins that mediate the recognition of pathogen associated molecular patterns (PAMPS) [22]. The TLR family of receptors is composed of up to 10 members in humans and 12 in mice [23]. TLR4 is the mammalian receptor recognizing bacterial lipopolysaccharide (LPS), the main cell wall component of Gram-negative bacteria, and plays a critical role in sepsis and controlling bacterial infections [24]. The molecular basis of how TLR4 mediates activation of RAS-GTPase upstream to MEK is not precisely understood. Association of LPS with MD2 as well as other lipid based mediators (e.g. lipid A) interacts with TLR4 to recruit several adaptor proteins [25]. This is thought to lead to the subsequent activation of receptor tyrosine kinases (RTKs) and the classical RAS-RAF-MEK1/2 cascade [26–28]. Additionally, TLR4 ligation can activate ERK1/2 through alternative pathways [29,30]. For instance, LPS can activate MEK/ERK through PKC ζ [31]. However, the differential role of MEK isoforms in TLR4 mediated ERK activation is not well studied. In the study of Bouhamdan et al. we addressed the physiological function of MEK1 in macrophages and interrogated the role of the MEK1/2 isoforms in ERK1/2 activation in response to diverse stimuli, including TLR4 ligand, recombinant (r) IL-10 and retinoic acid (RA) [32]. Although previous studies extensively evaluated the role of MEK/ERK using conventional inhibitors, we evaluated the effect of MEK1 deficiency on the cellular response to TLR4 stimulation in macrophages using a genetic approach. Interestingly, we found virtually no ERK1/2 phosphorylation following LPS treatment in MEK1 deficient macrophages despite the presence of MEK2, suggesting a predominant role for MEK1 in TLR4 mediated activation of ERK1/2. Furthermore, MEK1 deficiency led to differential regulation of IL-12 and IL-10 production in response to LPS. Moreover, MEK1 deficiency was associated with increased activation of the signal transducer and activator of transcription (STAT) 4, a major transcription factor for IL-12 production [33]. However, when MEK1 deficient macrophages were challenged with LPS in the presence of rIL-10 or RA, it facilitated ERK1/2 phosphorylation and led to decreased STAT4 phosphorylation and lower IL-12 production. Our findings indicate that engagement of upstream signals differentially regulate MEK1 and MEK2 activation leading to various biological effects. Neither rIL-10 and RA alone nor TLR4 ligation evoked ERK activation in MEK1 deficient macrophages. This implies that in the absence of MEK1, ERK activation requires a collaboration of different kinases to overcome MEK1 deficiency. Our results also suggest that LPS stimulation alone cannot activate ERK1/2 through MEK2 in the absence of MEK1 unless additional receptors are engaged (e.g. IL-10 receptor or the RA receptor). One previous study in T cells showed that development of IL-10-producing T helper 1 cells requires repeated T cell receptor (TCR) ligation and a sustained ERK1 and ERK2 phosphorylation, suggesting that signal strength dictates ERK activation and its biological effect [34]. It remains unclear whether a stronger signal is able to recruit different kinases besides MEK.

MEK1 deficiency led to an increase in STAT4 tyrosine phosphorylation and increased IL-12 production. Our observations confirm that STAT4 is an important positive regulator of IL-12 and that ERK activation is important for IL-10 production [35–37] and show the essential function for MEK1 in macrophages in regulating the ERK1/2 and STAT4 pathways in response to TLR4 activation. The results of our study could lead to the assumption that MEK2 phosphorylates ERK1/2 in response to LPS in the presence of RA or rIL-10. However, it has been shown that ERK1 and ERK2 can be activated in a MEK- independent fashion in neuronal cells as well as immune cells including neutrophils [38]. It is possible that RA or rIL-10 activate kinases other than MEK2 that contributed to ERK phosphorylation. Candidate kinases that can activate ERK independent of MEK include PKC and PKA [21,38]. If these kinases activate ERK independent of MEK, specifically MEK2 in our case, it poses the question what is the physiologic role of MEK2?

To add another layer to the complexity, ERK regulates expression and function of several phosphatases including MKP-1 (DUSP-1), and PTEN as well as protein phosphatase 2A (PP2A) [6–8,39]. Through dephosphorylation, these phosphatases regulate the activity of various kinases including MEK/ERK and STAT proteins. In our work the lack of MEK1 led to increased STAT4 phosphorylation even in the absence of any stimulation. This may suggest a lack of negative regulation through a phosphatase, for instance PP2A.

Classically, RA acts as ligand-inducible transcription factor by binding as heterodimers with the retinoid X receptors (RXRs) to RA response elements (RAREs) located in regulatory regions of target genes [40]. In recent years, it has been shown that RA can induce rapid and transient MAPK activation, including ERK [41]. The effect of RA appears to be cell type specific, in some cells leading to a slow (up to hours) activation of ERK, whereas in other cell types, for instance bronchial epithelial cells to a rapid ERK activation [42]. Similarly, RA activates other kinases, including MEK, PKC, PKA and RSK as well as phosphatidylinositol-3-kinase and even p38 [42,43]. Such activation appears to depend on a non-genomic action of RA is mediated through receptors located at the cytoplasmic side of the cell membrane [43]. In our study RA, in the absence of LPS, did not evoke activation of ERK in MEK1 deficient macrophages, whereas the combination of RA and LPS led to a robust and rapid ERK phosphorylation followed by an increase in IL-10 production. Additionally, it is well known that IL-10 regulates the JAK-STAT and ERK pathways as well as negatively regulates IL-12 production [34,36]. Yet, it is not clear whether IL-10 mediated ERK phosphorylation in our study is due to the activation of the classic RAS-RAF-MEK pathway or mediated through alternative pathways.

**Figure 1:**
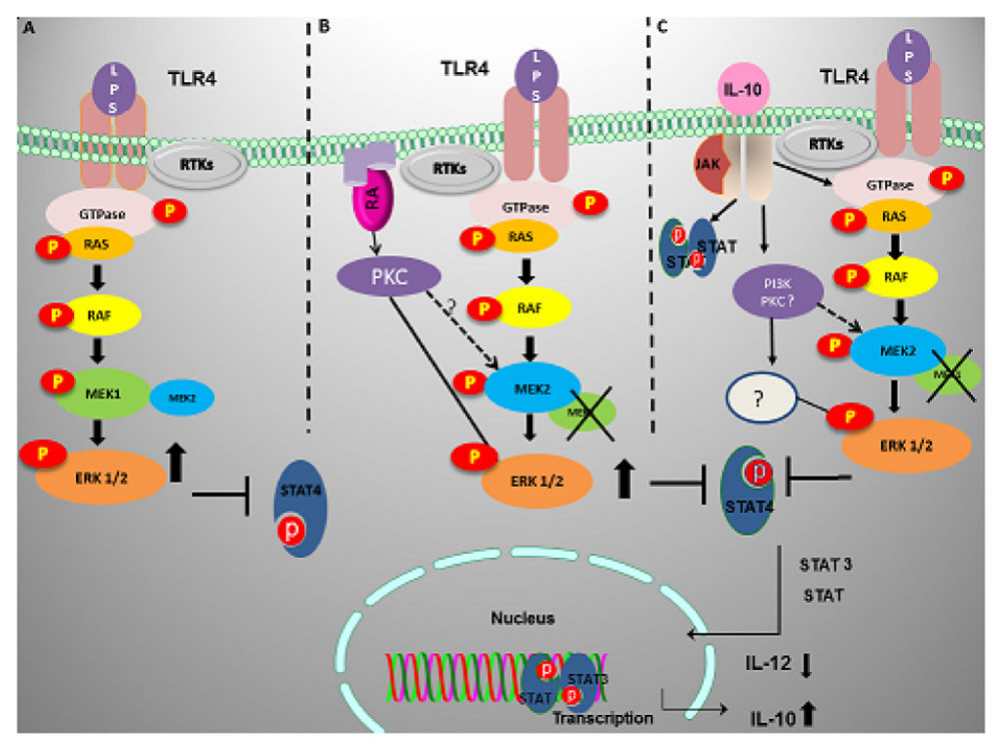
Schematic diagram for MEK1-dependent and independent ERK activation. **A.** In the presence of MEK1and MEK2, TLR4 engagement activates RTKs as well as RAS GTPase. This leads to activation of the RAF pathway upstream to MEK1/2, utilizing predominantly MEK1 to subsequently phosphorylate ERK1/2. **B.** In the absence of MEK1, ERK activation requires two signals. TLR4 (LPS) and RAR (RA) engagement may lead to co-activation (stronger signal) of the classical pathway through RTKs, RAS GTPase-RAF-MEK2 to activate ERK1/2. Alternatively, ERK can be activated directly by PKCζ. C. Co-activation of the TLR4 and IL-10 receptor (IL10R) leads to the activation of the classical RTKs, RAS GTPase-RAF pathways with subsequent activation of MEK2, followed by phosphorylation of ERK1/2. Alternatively, co-stimulation of TLR4 and IL10R may activate PI3K and/or PKC followed by activation of ERK in the absence of MEK1 either through MEK2 or through the activation of unknown kinases. Engagement of IL-10 to its receptor activates the JAK-STAT pathway, predominantly STAT3 with positive feedback on IL-10 production. Similarly, activation of ERK parallels a decrease in STAT4 phosphorylation but an increase in STAT3 phosphorylation resulting in decreased IL-12 promoter activity.

Although, our work shed some light on the role of TLR4 mediated MEK/ERK activation it raises several unanswered questions that need further investigation, especially the role of MEK2 in macrophages and innate immunity as well as the potential alternative pathways in regulating ERK activation in the absence of MEK1 or MEK2. Proposed pathways leading to ERK activation in the absence of MEK1 in response to a combination of LPS and RA or LPS and IL10 are shown in (Figure 1).
